# Nicotine-mediated therapy for Parkinson’s disease in transgenic *Caenorhabditis elegans* model

**DOI:** 10.3389/fnagi.2024.1358141

**Published:** 2024-05-15

**Authors:** Inam Ullah, Longhe Zhao, Shahab Uddin, Yangtao Zhou, Xin Wang, Hongyu Li

**Affiliations:** ^1^School of Life Sciences, Lanzhou University, Lanzhou, China; ^2^School of Pharmacy, Lanzhou University, Lanzhou, China; ^3^Department of Neurology, Clinical Center for Parkinson's Disease, Xuanwu Hospital of Capital Medical University, Beijing, China

**Keywords:** Parkinson’s disease, nicotine, α-Synuclein, 6-hydroxydopamine, *Caenorhabditis elegans*

## Abstract

Parkinson’s disease resultant in the degeneration of Dopaminergic neurons and accumulation of α-synuclein in the substantia nigra pars compacta. The synthetic therapeutics for Parkinson’s disease have moderate symptomatic benefits but cannot prevent or delay disease progression. In this study, nicotine was employed by using transgenic *Caenorhabditis elegans* Parkinson’s disease models to minimize the Parkinson’s disease symptoms. The results showed that the nicotine at 100, 150, and 200 μM doses reduced degeneration of Dopaminergic neurons caused by 6-hydroxydopamine (14, 33, and 40%), lowered the aggregative toxicity of α-synuclein by 53, 56, and 78%, respectively. The reduction in food-sensing behavioral disabilities of BZ555 was observed to be 18, 49, and 86%, respectively, with nicotine concentrations of 100 μM, 150 μM, and 200 μM. Additionally, nicotine was found to enhance Daf-16 nuclear translocation by 14, 31, and 49%, and dose-dependently increased SOD-3 expression by 10, 19, and 23%. In summary, the nicotine might a promising therapy option for Parkinson’s disease.

## 1 Introduction

A debilitating neurodegenerative disease associated with aging, Parkinson’s Disease (PD) is second after Alzheimer’s Disease (AD) (Reich and Savitt, 2019). A study of PD prevalence was arise by 1 and 5%, in the age of 65 and 85-year-olds individuals, respectively. In addition, its prevelance is more common in men than women (Pringsheim et al., 2014; Simon et al., 2020). A British physician named James Parkinson first named Parkinson’s disease “shaking palsy” in 1817. It was renamed Parkinson’s disease by Jean-Martin Charcot in June 1988 (Przedborski, 2017; Jankovic and Tan, 2020). The disease progresses mainly due to the loss of dopaminergic neurons in the substantia nigra (SNPc) of the midbrain and α-synuclein (α-syn) aggregation in the brain tissues (Polymeropoulos et al., 1997; Spillantini et al., 1997; Du et al., 2020). Although PD is mainly characterized as a movement disorder, non-motor features are also present. Motor impairment includes resting tremor, rigidity, postural imbalance, slowness of movement, shuffling gate, and lack of facial expression (Schilder et al., 2017; Wei et al., 2017), while non-motor impairment includes constipation, dysphagia, hallucination, behavioral and cognitive problems in the disease late stages including sleep disturbances, anxiety and dementia (Titova and Chaudhuri, 2017; Muhammad et al., 2022). There are numerous theories explaining how Parkinson’s disease manifests or the progression of its symptoms even though the exact cause is unknown (Chagas et al., 2014).

The etiology of PD advancements is due to a complex interaction of both sporadic (90% of cases) and genetic (5–10%) aspects (Blauwendraat et al., 2020). It is possible to develop sporadic Parkinson’s disease due to exposure to toxicants, free radicals, head trauma and inflammation of the nervous system (Singleton and Hardy, 2019; Ullah et al., 2021). There are various genes associated with genetic predisposition including α-syn, parkin (PRKN), leucine-rich repeat kinase 2 (LRRK2) and PTEN-induced kinase 1(PINK1) (Bandres-Ciga et al., 2020). The primary barrier in PD diagnosis and prognosis is that its signs and symptoms do not appear before 70–80% of brain substantia nigra pars compacta (SNPc) region DA neurons degeneration. United States food and drug administration (FDA) approved drugs mere delay and alleviate disease symptoms and do not stop, reduce or reverse disease progression (Sivanandy et al., 2021; Zhang et al., 2021). In the researcher’s quest for pharmacological research, new approaches are developed to control the emergence, stop progression, and eliminate the disease. Therefore, scientists are optimistic about discovering a treatment for Parkinson’s disease using neuroprotective drugs based on component-based molecules (van der Perren et al., 2020; Detroja and Samson, 2022).

**Figure 1:**
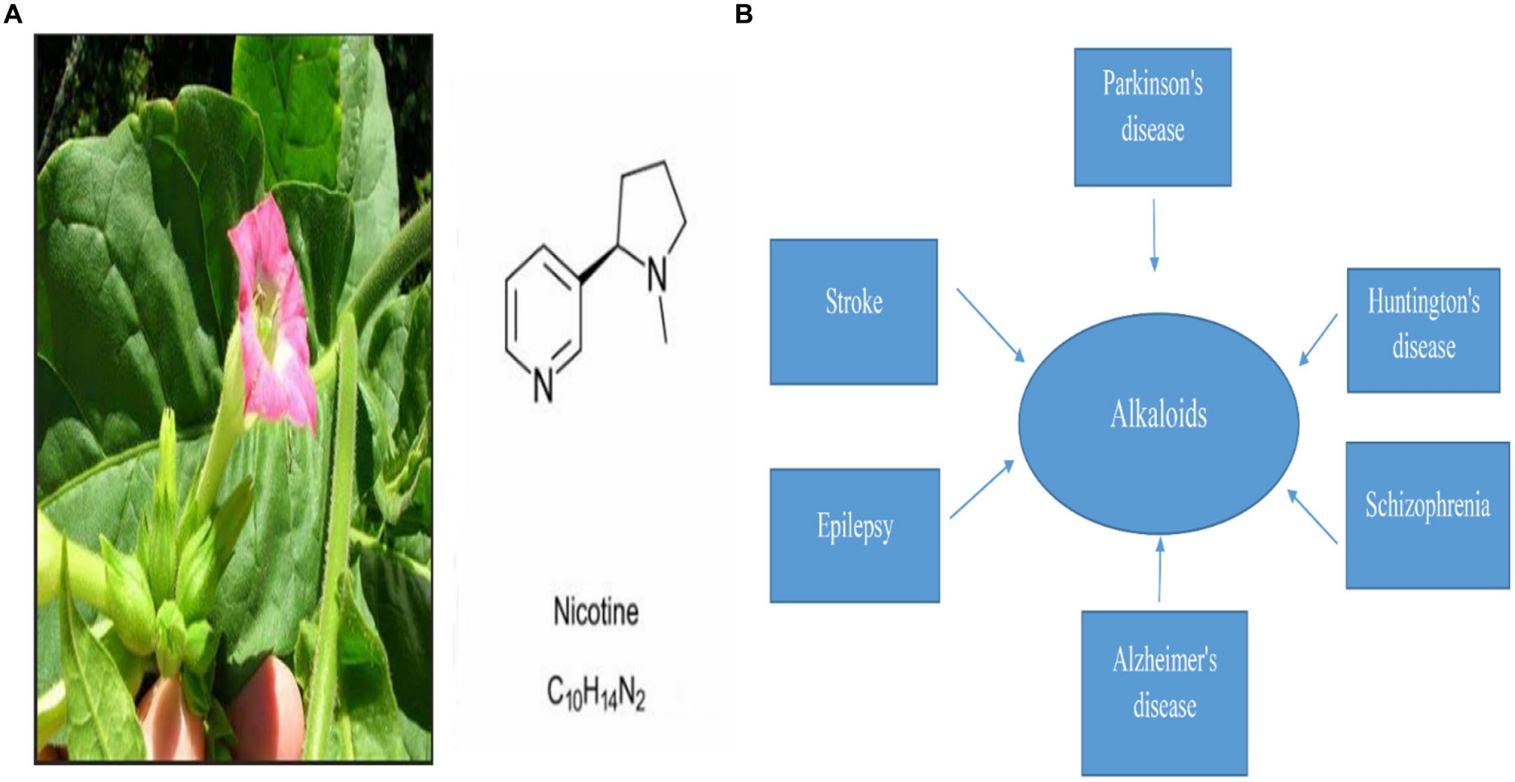
**(A)** Represents Nicotine’s chemical structure and its plant source, *nicotiana tabacum*, and **(B)** represents Alkaloid’s pivotal role in the treatment of different neurodegenerative diseases (Dimatelis et al., 2012; Cianciulli et al., 2022; Sokouti et al., 2022; Wang et al., 2022; Zahedipour et al., 2022).

*Nicotine* is a natural pyridine alkaloid found mainly in *Nicotiana tabacum* of the Solanaceae family Figure 1. Nicotinic acetylcholine receptor stimulation is the main mechanism of its pharmacological properties in the central and peripheral nervous systems (Ribeiro and Leite, 2003). Several studies have found that *nicotine* improves synaptic plasticity and dopaminergic neuronal survival. As well as reducing neuroinflammation and mitochondrial dysfunction, it also inhibits oxidative stress (Hussain et al., 2018; Nourse et al., 2021). It has been shown that smoking is inversely related to PD incidence in more than 40 epidemiological studies conducted in the past 50 years (Quik et al., 2012; Ma et al., 2020). There are several studies that suggest *nicotine* may reduce the prevalence of Parkinson’s disease (Migliore and Coppedè, 2009; Nicholatos et al., 2018; Getachew et al., 2019).

Therefore, we evaluated *nicotine’s* neuroprotective properties in transgenic *C. elegans*. In this study, nicotine reduced α-syn accumulations and restored 6-OHDA intoxicated DA neurons in the transgenic *C. elegans* PD models. Nicotine also restored the food-sensing behavior of 6-OHDA-intoxicated worms and improved lipid content and dopamine levels. Moreover, *nicotine* enhanced the antioxidant activities of superoxide dismutase-3 (SOD-3) and FOXO transcription factor homolog (Daf-16) nuclear translocations in the transgenic *C*. elegans model. As a result, we consider nicotine to be both an adjuvant and a prophylactic agent in treating Parkinson’s disease using the transgenic *C. elegans* model system.

## 2 Materials and methods

### 2.1 *Caenorhabditis elegans* strains, culture, maintenance, and synchronization

With its transparent body, short life span, easy culture methods, ease of maintenance, simple neuronal network, conserved neuronal pathways, and robust genetic screens for investigating molecular mechanisms, *C. elegans* makes an attractive model in various biological disciplines (Kleawyothatis et al., 2023; Yuan et al., 2023). Table 1 lists the C. elegan strains investigated in our study to assess Nicotine’s therapeutic potential for PD including *N2 (Wild-type nematode), OW13* (*pkIs2386, unc-*54p::human α-synuclein:: YFP + unc-119(+), BZ555) (egIs1, dat-1p::GFP; green fluorescent protein (GFP)), TJ356 (zls356 [daf-16p::da16a/b::GFP + rol-6(su1006)]), CF1553 ([muIs84 ((pAD76) SOD-3p:: GFP + rol-6)]). All *C. elegans* were purchased through the Caenorhabditis Genetics Centre (CGC), Minnesota, United States. We maintained the worms in solid NGM (nematode growth media) at 20°C and fed them *E*. coli (OP50) bacteria strains as food. Previously described maintenance procedures were followed for *C. elegans* (Fu et al., 2014; Muhammad et al., 2022). By treating gravid adults with hypochlorite (0.5 M NaOH and 2% sodium hypochlorite), fertilized eggs were obtained. To obtain the desired larval stage to treat with nicotine in the various experiments given below, synchronized eggs were placed onto OP50-seeded fresh NGM plates or liquid M-9 and incubated at 20°C for 24 h.

**Table 1:** Study strains and maintenance conditions for *C*. elegans.

Strains	Transgene’s	Tem (°C)	Phenotypes
N2	Wild-type nematode	20	Wild type movement
OW13	pkIs2386, unc-54p::humaEEn α-synuclein:: YFP + unc-119(+); yellow fluorescent protein (YFP) expression in the muscles.	20	Muscular α-synuclein expression
BZ555	egIs1, dat-1p::GFP; green fluorescent protein (GFP)	20	Dopaminergic Neurotoxic model
TJ356	zls356 [daf-16p::da16a/b::GFP + rol-6(su1006)]	20	GFP for Daf-16 pathways expression
CF1553	[muIs84 ((pAD76) SOD-3p:: GFP + rol-6)];	20	Anti-oxidative enzymes expressions

### 2.2 Chemical reagents

Nicotine (Lot#DST180802-032, CAS#54–11-5, HPLC≥98%) was purchased from DESITE (China), 6-hydroxydopamine hydrobromide (6-OHDA; Lot#MKCC1473) was purchased from Sigma-Aldrich (USA), L-Ascorbic acid (Lot. QGNMA-RN, CAS 50–81-7 > 99.0%) purchased from T.C.I. chemicals (Shanghai, China), 2’-Deoxy-5-fluorouridine (FUDR) Lot.#SYEWM-OF, CAS#50–91-9 > 98.0% was purchased from T.C.I chemicals (Shanghai, China), Enzyme-linked immunosorbent assay (ELISA) kit for dopamine (DA) Lot#L190628620 was purchased from Cloud-Clone Corp (China), Juglone (cas#481–39-0, lot#1104A025 (Solar bio), 2,7, dichlorofluorescein diacetate HPLC≥95% cas#2044-85-1, lot#BCBZ7835) was purchased from Sigma life science Germany.

### 2.3 Food clearance assay

In this assay *C. elegans* strains OW13 and BZ555 were tested to assess nicotine’s effect on their physiology and determine nicotine’s appropriate dosage concentrations for experiments in the future (Ru-Huei et al., 2014; Chalorak et al., 2021). After overnight growth, *E*. coli was resuspended in S-medium at an optimum density (O.D.) of 6.6. Nicotine’s desired concentration was achieved by diluting the drug in an *E. coli* suspension. A 150 μL volume of the final mixture was added per well in the 96-well plate, an approximately synchronized group of 20–30 L1 animals in 10 μL s-medium was added to an *E*. coli suspension containing a series of nicotine concentrations, which was incubated for six days in 96-well plates at 25°C. In order to prevent solution evaporation, aluminum foil was used to cover the plates. A Microplate Reader M2 SpectraMax (Molecular Devices, Silicon Valley, CA, United States) was used to measure the OD for six days at 600 nm once/day. Before measuring the OD, each plate was placed on a plate shaker for 10 s. A microscopical analysis determined the fraction of live worms in the wells based on size. Each experiment was repeated three times.

### 2.4 The self-induction of da neurodegeneration in transgenic *Caenorhabditis elegans* by 6-OHDA

BZ555 (GFP-tagged Dopaminergic Neurotoxic model) *C. elegans* neurons were subjected to neurotoxin (6-OHDA) to stimulate self-neurodegeneration. Briefly, with some minor alterations to the previously described protocol (Nass et al., 2002; Thirugnanam and Santhakumar, 2022). We added 100 mM 6-OHDA and 20 mM ascorbic acid to OP50/S-medium mix both with and without nicotine. *BZ555* worms at L-3 (~200 in number) moved into the treated groups, incubated at 22°C for 1 h, and jiggled slightly every 10 min to induce neurodegeneration. After an incubation period of 1 h, BZ555 were washed three times with washing buffer (M9), moved to the OP50/NGM plates with or without nicotine different concentrations, and incubated again for 72 h at 20°C. OP50/NGM/nicotine plates containing 0.06 mg/mL FUDR (5-fluoro-29-deoxyuridine) (Sigma, and St. Louis, MI) because FUDR stops eggs from hatching in *C. elegans* and thus reduceses its population on plates. Each experiment was repeated three times.

### 2.5 Quantitative assay of dopaminergic neurons degeneration

The DA neurons degeneration assay was performed in BZ555 *C. elegans* treated with or without 6-OHDA/nicotine at various concentrations following a previously described protocol with a few modifications (Hibshman et al., 2021). Following 72 h of incubation at 20°C, strains of *BZ555* were washed three times with M9 buffer to remove adhesive bacteria, mounted on glass slides, immobilized with 100 mM sodium azide, enclosed with a coverslip, and monitored under fluorescence microscope. The BX53 microscope (Olympus Corp., Tokyo, Japan) was used to observe immobilized fluorescent images of *BZ555* C elegans. Quantifying fluorescence intensity was done using ImageJ software. Each experiment was repeated three times.

### 2.6 An assay for measuring dopamine levels

We used an Enzyme-linked immunosorbent assay (ELISA assay) kit to evaluate dopamine content in 6-OHDA and 6-OHDA+nicotine’s different concentrations treated *C. elegans* groups (Bi et al., 2016). In each group, 6-OHDA treated and 6-OHDA/nicotine treated *C. elegans BZ555* L3 larvae were washed three times with M9, then weighed. Then, according to the protocol, we added 100 μL, 1X RIPA+Protease inhibitor and used the Dounce homogenizer on ice for complete lysis of the worms. The worms were centrifuged at 4°C for 10 min at 14,000 × *g*. In accordance with the kit standard protocol, the supernatant was centrifuged and then analyzed by ELISA for DA content. Each experiment was repeated three times.

### 2.7 Quantitative assay of α -synuclein accumulation

It is thought that the protein (α -Syn) in *OW13* is one of the primary causes of PD. As a result, we chose *OW13* (GFP-tagged Muscular α-synuclein expression), a transgenic *C. elegans* PD model. OW13 worms integrated with human α-syn protein plus GFP fusion construct expressed α-Syn in the muscle’s walls via green fluorescence signals. The method described previously has been modified slightly (Liu et al., 2015; Wang et al., 2020). A synchronized larva stage1 (L-1) strain of C. elegans was transferred onto NGM plates with or without nicotine at 100, 150, and 200 μM and seeded with OP50 and incubated at *20°C* for 72 h. 0.06 mg/mL FUDR was added to each NGM drug treated and untreated plates. Nicotine was not administered to control groups. Worms were washed thrice with M9 buffer, mounted on a glass slide, immobilized with sodium azide 100 mM, and enclosed with coverslips after 72 h. Following that, we analyzed α-syn green fluorescent protein (GFP) expression intensity in the treated and untreated worms under the BX53 fluorescent microscope (Olympus Corp., Tokyo, Japan). Fluorescence intensity was quantified using ImageJ software. Each experiment was repeated three times.

### 2.8 Lipid deposition quantification by Nile red staining assay

For quantitative lipid deposits analysis, in *OW13* we used intracellular lipid droplets detective specific fluorescent staining dye Nile red. For stock solution preparation the previously described protocol has been modified slightly (Muhammad et al., 2022). We prepared a 0.5 mg/mL stock solution with 0.5 mg Nile red dye dissolved in 1 mL acetone. As a next step, the stock solution was mixed with *E. coli* in 1:250, then spread over the NGM plates containing different nicotine concentrations along with 0.06 mg/mL FUDR, and incubated overnight at 37°C. A further 72 h incubation was performed for L-1 *C. elegans* on NGM/Nile Red/OP50/FUDR plates with or without nicotine at 100,150, and 200 μM. Wild-type N2 *C. elegans* was used as a negative control. After 72 h, the worms were washed three times with M9, mounted on a glass slide, immobilized with sodium azide 100 mM, and enclosed with coverslips. The fluorescence intensity of lipid deposition was measured in *C. elegans* using a fluorescence microscope (Olympus, BX53, Japan) and the intensity was quantified using ImageJ software. Each experiment was repeated three times.

### 2.9 Food-sensing behavior assay

There is evidence that dopamine is responsible for *C*. elegans’ basal slowing response (de Guzman et al., 2022). To evaluate DA neuron integrity, food-sensing behavior assays were performed. When *C. elegans* are surrounded by food sources, they move slower than when they are without them. We modified the previously described protocol slightly to prepare the NGM plates (Malaiwong et al., 2019). Two types of NGM plates were designed, one with food source (*E. coli*) and one without (*E. coli*). Following intoxication with 6-OHDA alone, and 6-OHDA+different nicotine concentrations for 1 h at 20°C, with 10 min intervals of mixing, the BZ555, L3 larva were washed three times with M9. A total of 30–50 *C. elegans* were placed in 10 μL M9 on NGM plates with and without bacterial lawns. In both bacterial and non-bacterial lawns, locomotory rate of the worms was assessed after 5–10 min of transfer by counting body bending at 20-s intervals. By calculating percentages of locomotion rates in bacteria lawns over non-bacterial lawns, the slowing rate was estimated. We calculated and compared the average slowing rates of 15–20 worms in each group for each analysis. Each experiment was repeated three times.

### 2.10 *In-vivo* oxidative free radicals analysis

A previously described protocol with minor modifications was used in order to evaluate the level of free oxidative radicals *in-vivo*, using 2,7-dichlorofluorescein diacetate (H2DCF-DA, Sigma) (Pandey et al., 2019). A synchronized population of L1 larvae of OW13 was cultured on nicotine-treated and untreated NGM plates for 72 h at 20°C. C elegans were washed three times with M9 after 72 h. 50 *C. elegans* were shifted to 96-well plates in 150 μL of phosphate buffer solution (PBS,) and 150 μL of 100 mM H2DCF-DA was added per well before reading. Using a microplate reader at 485 nm excitation and 570 nm emission, the fluorescence reading was taken after 2 h. Each experiment was repeated three times.

### 2.11 Thermotolerance assay

Based on the protocol previously described, a thermotolerance assay was performed with slight modifications (Yang et al., 2018). On the nicotine-treated and untreated NGM plates, we cultured approximately 50 synchronized *OW13*, L1 larvae for 7 days at 20°C. Incubation at 37°C for 3 h was started on day 7 of the experiment. Touching each worm with platinum wire every hour was used to score the dead and alive worms. Each experiment was repeated three times.

### 2.12 Nicotine oxidative stress resistance assay

In the BZ555 strain, we measured nicotine oxidative stress resistance using Juglone (5-hydroxy-1,4-naphthoquinone), an intracellular reactive oxygen species (ROS) generator using the previously mentioned protocol with modifications (Reigada et al., 2020). Synchronized L1 of BZ555 was exposed to 100,150,200 μM of our drug nicotine for 72 h. at 20°C. Control group is BZ555 with without nicotine treatment. After 72 h, the *C. elegans* were washed three times with M9. 50 μL M9 containing 40–50 *C. elegans* were poured into 96 wells, and various drug concentrations and 250 μM Juglone were added. Incubated for 24 h. A touch-provoked movement was used to assess the survival percentage of worms after 24 h. Responses to the mechanical stimulus were counted as alive, and vice versa. Each experiment was repeated three times.

### 2.13 Daf-16 nuclear translocation assay

A previously described protocol, modified slightly, was used to determine the effect of nicotine on the nuclear localization of DAF-16 in *TJ356 zls356 [daf-16p::da16a/b::GFP + rol-6(su1006)] (DAF-16:: GFP fusion protein)* transgenic L1 worms (Lee et al., 2018). Synchronized L1 larvae were put on *nicotine*-treated/untreated NGM plates incubated for 72 h at 20°C. In the following steps, *C. elegans* were washed three times with M-9 buffer, mounted on a glass slide containing 100 mM sodium azide, and covered with a coverslip. DAF-16::GFP nuclear localization was observed under a fluorescence microscope (Olympus BX53) based on the discrete fluorescence aggregation phenotype. ImageJ software was used for analyzing the images (Carl Zeiss, Gottingen, Germany). Each experiment was repeated three times.

### 2.14 Sod-3 expression assay

*CF1553([muIs84 ((pAD76) SOD-3p:: GFP + rol-6)];)* a transgenic strain expressing SOD-3::GFP, was used for the reporter gene assay. To study nicotine’s effect on CF1553 strains, the previously described protocol with modifications was used (Yang et al., 2018). The L-1 synchronized *C. elegans* were transferred to solid media plates with or without nicotine at 100, 150, and 200 μM and incubated for 72 h at 20°C. Nicotine is not administered to the control group. In the following steps, *C. elegans* were washed three times with M-9 buffer, mounted on a glass slide containing 100 mM sodium azide, and covered with a coverslip. The fluorescence intensity of the treated group’s images was observed using a fluorescence microscope (Olympus, BX53, Japan). ImageJ was used to calculate the fluorescence intensity of *C. elegans* images. Each experiment was repeated three times.

### 2.15 Statistical analysis

We used SPSS/ANOVA (one-way), ImageJ, and GraphPad PRISM, Version 8.02 (1992–2019 GraphPad Software, Inc., La Jolla, CA, United States) for statistical analysis. DA neurons’ GFP intensities were calculated as means ± SD, and their *p*-values were calculated. Likewise, for α-synuclein, the means ± SD of YFP intensity was also calculated. Each experiment was repeated at least three times and normalized to control groups. Significance differences (p-values) were observed via SPSS/(one-way) ANOVA, followed by Tukey’s or Dunnett’s (where comparison was only to the untreated group) post-hoc tests. In all the experiments, *p* ≤ 0.001 to *p* ≤ 0.005 is considered statistically significant and represented by (p ≤ 0.005)*.

## 3 Results

### 3.1 Nicotine effect on transgenic *Caenorhabditis elegans*

**Figure 2:**
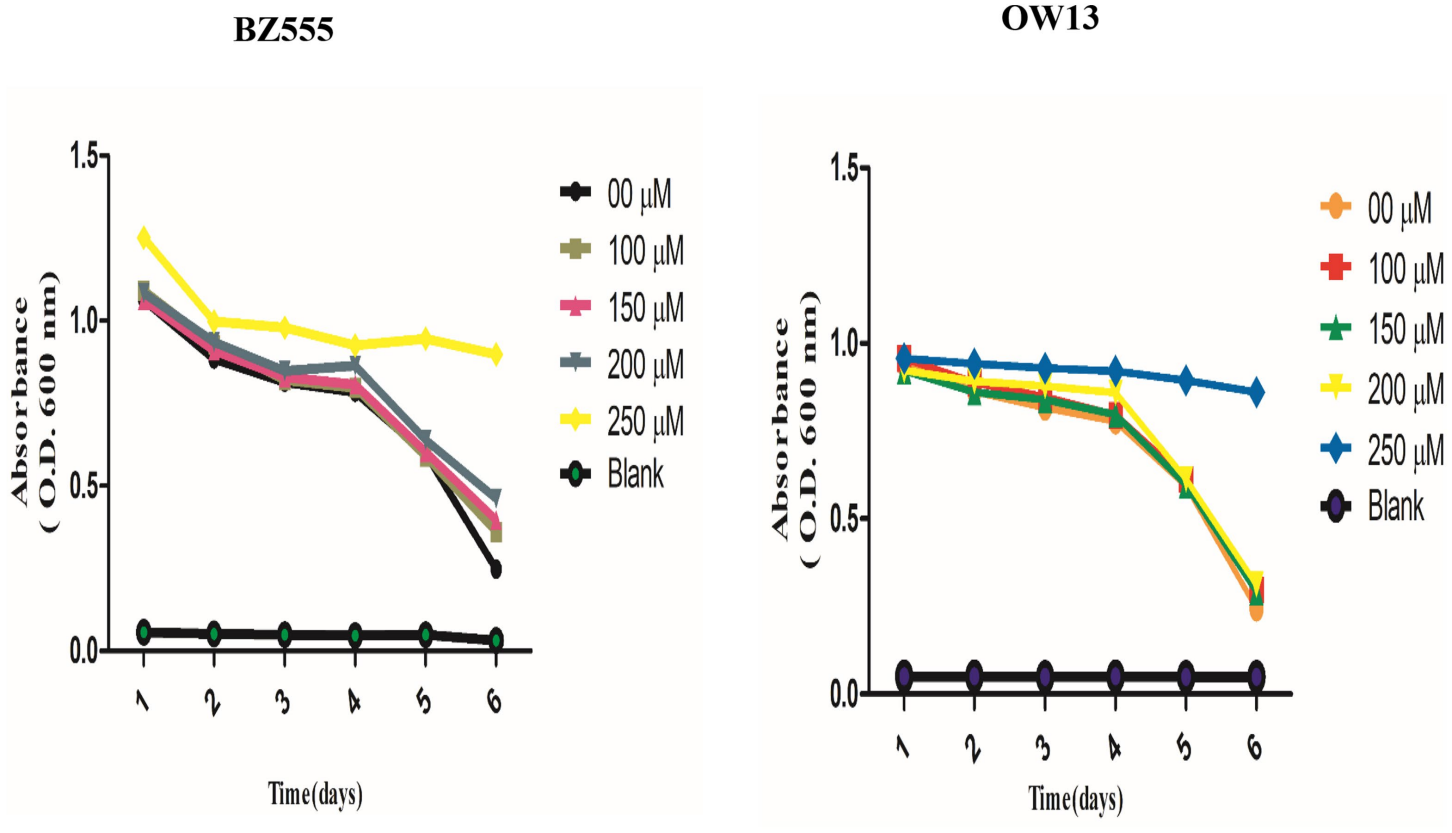
Explaining the non-toxic concentration outcomes of nicotine food clearance tests in *C. elegans* PD models. Approximately 30 L-1, synchronized *C. elegans* OW13, and BZ555 were cultured at 25°C in a 96-well microtiter plate with OP50 suspensions (OD 600 nm) and nicotine at 0 μM, 100 μM, 150 μM, 200 μM, and 250 μM dilutions. The OD values were calculated once a day for 6-days. The absorbance (600 nm) was determined by using SpectraMax M2 Microplate Reader. Worms treated at 100 μM, 150 μM, and 200 μM nicotine were alive. But, the worms treated at 250 μM dilutions started dying daily. The significant difference between nicotine dilutions in treated models (BZ555 and OW13) is measured *p* ≤ 0.005 (***).

We performed a food clearance test to assess nicotine toxic concentrations in *C. elegans*. We treated transgenic (OW13) and pharmacological (BZ555) worm strains with nicotine at 100, 150, 200, and 250 μM. A concentration of nicotine was tested to determine whether nicotine would be non-toxic to the physiology and hatching capabilities of *C. elegans*. In our experiment, worms treated with nicotine at 250 μM dilution showed toxic behavior, resulting in smaller bodies and fewer offspring. Even adults die after day intervals. Therefore, it lowers the clearance curve for food. The rest of the treated groups were alive and in good physical health. The addition of nicotine to cultures containing BZ555 and OW13 at 100, 150, and 200 μM concentrations did not affect food clearance compared to 250 μM Figure 2. In our subsequent experiments, we used the highest concentration of 200 μM.

### 3.2 Nicotine diminished 6-OHDA intoxicated DA neuron degeneration in a dose-dependent manner

**Figure 3:**
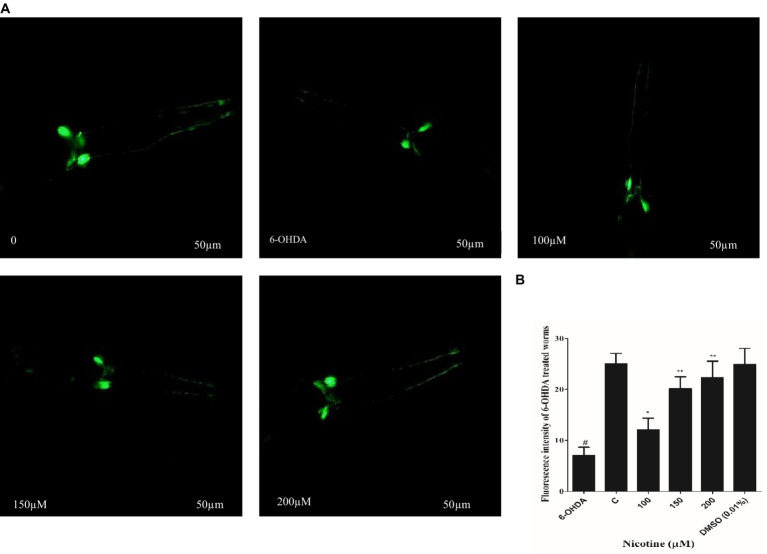
Represents nicotine attenuates the degeneration of DA neurons by 6-OHDA intoxication in BZ555 transgenic *C. elegans*. **(A)** Images representing fluorescence GFP: Pdat-1 expression patterns in DA neurons of the worms BZ555. **(B)** Graphical demonstration of GFP expression pattern of fluorescence in DA neurons of the worms BZ555 treated with or without 6-OHDA/nicotine was computed using ImageJ software. Scale bar 50 μm. The results indicate nicotine at 100,150, and 200 μM recovers the DA neuron degenerations up to 14, 33, and 40% dose-dependently. Data were computed by mean ± SD (*n* = 30). A hash (#) indicates significant differences between 6-OHDA-treated and untreated animals (< 0.005).

*C. elegans* possesses eight DA neurons, comprising one pair of anterior deirid (ADE) neurons, two pairs of cephalic (CEP) neurons in the head position, and one pair of posterior deirid (PDE) neurons in the posterior lateral region. *C. elegans* strain (BZ555) tagged with GFP at the dat-1 promoter is used as a PD model to study the active role of DA neurons. The promoter (Pdat-1: GFP) was used to observe significant changes in DA neurons’ GFP intensity in the head region of the worm. The neurotoxin 6-OHDA was used to generate selective degenerations of DA neurons, which mimicked one of the symptoms of PD in BZ555 *C. elegans*. To analyze nicotine efficiency, we evaluated neuronal integrity by quantifying the loss of expression of a GFP reporter gene in DA neurons of 6-OHDA-treated BZ555 animals. 6-OHDA treatment reduced GFP expression in PDE neurons and partially lost GFP expression in CEP and ADE neurons Figure 3A. Fluorescence intensity in 6-OHDA-exposed worms decreases by 93% (*p* ≤ 0.005) compared to non-exposed groups. When nematodes were subjected to nicotine, remarkable protection was observed in DA neurons with CEP, and ADE neurons showed enhanced GFP expression, providing evidence for nicotine’s neuroprotective effects. Furthermore, nicotine at 100, 150, and 200 μM enhanced the fluorescence intensity of GFP in 6-OHDA-intoxicated BZ555 by 14, 33, and 40%, respectively Figure 3B.

### 3.3 Nicotine reduced α-synuclein aggregation in transgenic *Caenorhabditis elegans* in a dose-dependent manner

**Figure 4:**
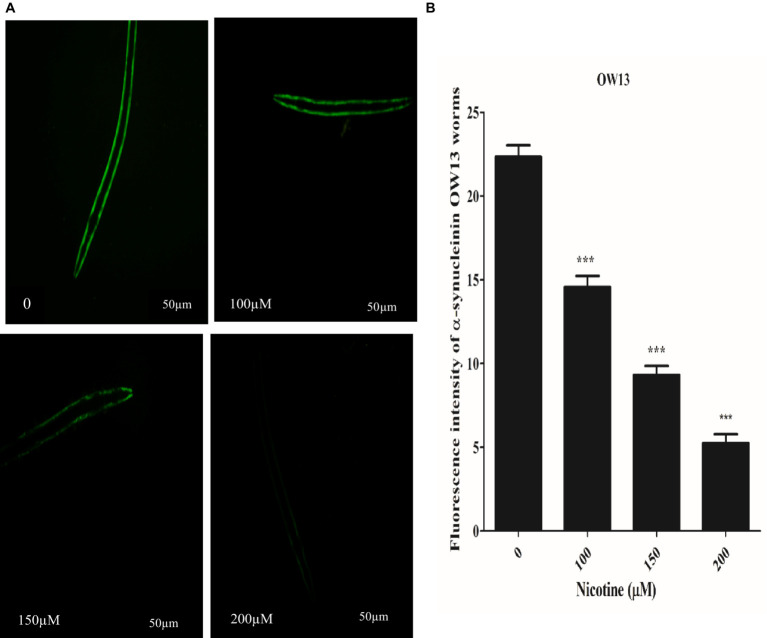
A represents that Nicotine treatment decreases α-Syn accumulations in OW13 worms. **(A)** GFP fluorescence expressions in OW13 muscle walls. **(B)** Graph representing the data of fluorescence intensities in worms calculated by mean ± SD (*n* = 30). Scale bar 50 μm. ImageJ software was used to quantify the fluorescence images. Data shows that nicotine at 100, 150, and 200 μM attenuated the α-Syn accumulations up to (53%), (56%), and (78%) (*p* ≤ 0.005) compared to untreated groups, dosedependently. (*) An asterisk depicts the significant difference between nicotine-treated and untreated groups (*p* ≤ 0.005).

*C*. elegans are devoid of the SNCA orthologous gene. In contrast, nematode genetic flexibility enables the transgenic overexpression of human SNCA genes in muscle walls to investigate SNCA accumulation (Holmqvist et al., 2014). We selected *C. elegans* OW13 transgenics for examining protein α-Syn aggregation. As a result of the strain’s advantages, OW13 displays higher expression levels and progressive motility defects like PD. Consequently, it demonstrated *in vivo* aggregative toxicity of protein α-Syn (Muhammad et al., 2022). To observe the active role of nicotine in decreasing α-Syn accumulated toxicity, we treated the worms with or without nicotine. Our assessment verified that nicotine at 100, 150, and 200 μM significantly reduced α-Syn aggregates fluorescence intensity by about 53, 56, and 78% (*p* ≤ 0.005) compared to untreated groups, dose-dependently. ImageJ software quantifies α-Syn numbers and aggregate volumes of fluorescence intensity Figure 4.

### 3.4 Nicotine recovers lipid deposition in transgenic strains *Caenorhabditis elegans*

**Figure 5:**
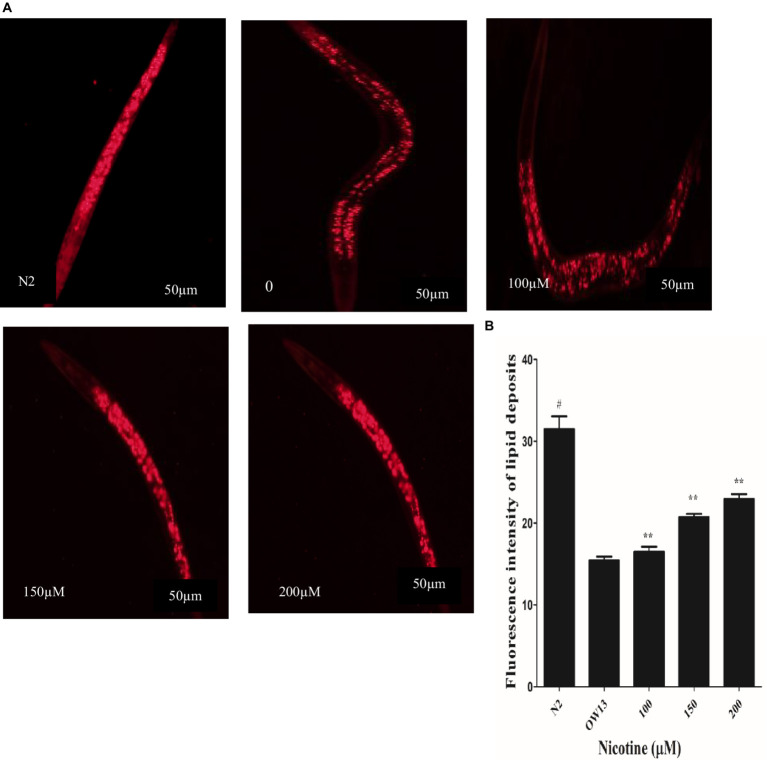
Nicotine increases lipid content in OW13 nematodes. **(A)** Representative Nile red staining images of nematode strains OW13 & N2. **(B)** Graphical representation of fluorescence intensities after Nile red staining as calculated by ImageJ. The data shows the mean ± SD (*n* = 30). Scale bar 50 μm. The N2 group was used as a negative control to verify whether α-Syn existence affects lipid deposits. The double asterisk (**) shows the significant augmentation of lipid deposits after nicotine treatment (6, 26, 33%) compared to the untreated groups (*p* ≤ 0.005), and hash (#) describes the significant difference between N2 and untreated group (OW13 without nicotine treatment) (*p* ≤ 0.005).

Protein α-Syn has associations with lipid contents and fatty acid modifications, and together they start vesicle formation via a definite mechanism (Jadiya et al., 2011). In OW13 worms, the lipid contents are significantly reduced due to α-Syn existence (Chalorak et al., 2018). α-Syn toxicity disrupted lipid composition in the worms. Moreover, the toxic nature of accumulated α -Syn leads to lipid peroxidation (Saewanee et al., 2021). Lipid molecules are not only responsible for supporting a cell’s structure and constructing membrane components, they are also involved in CNS communication. After nicotine treatment at different concentrations in OW13, we analyzed the lipid content in untreated and nicotine-treated OW13 strains through Nile red staining. In our experiments, nicotine increased the level of lipids by 6.0, 26, and 33% (*p* ≤ 0.005) dose-dependently Figure 5. A negative control, N2, was also used to compare the lipid content of transgenic OW13 with N2. The lipid content of N2 was 51% higher than that of untreated OW13.

### 3.5 Nicotine recovers food sensing behavior in transgenic strain *Caenorhabditis elegans* in a dose-dependent manner

**Figure 6:**
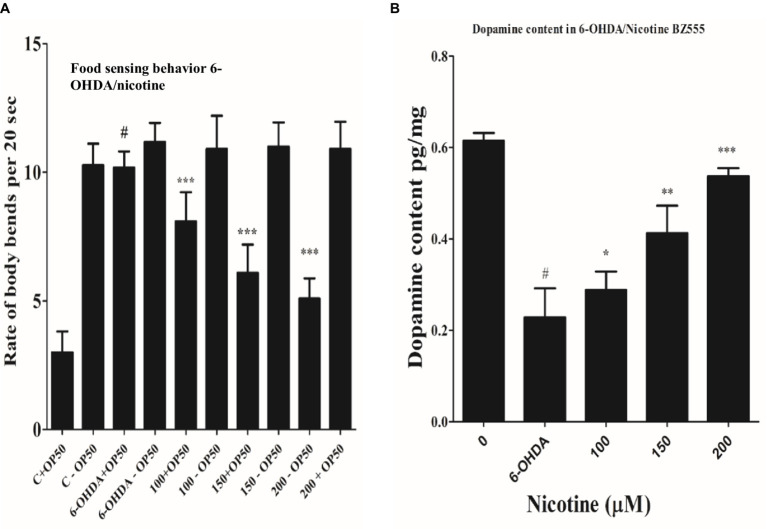
**(A)** Nicotine ameliorated DA neuron degeneration in 6-OHDA-treated BZ555 worms. The bending frequency (locomotory rate) of the groups 6-OHDA treated and un-treated, while 6-OHDA/nicotine-treated or un-treated *C. elegans* were in a bacterial lawn or without bacterial lawns assessed in this behavior test. Results exhibited that nicotine at 100 (18%), 150 (49%), and 200 (86%) in BZ555 diminished DA neuron degeneration. The data was calculated using the mean ± SD (*n* = 30). # hash represents a significant difference between control and 6-OHDA treated nematodes. In contrast, * asterisk represents the significant difference between the worm groups 6-OHDA/nicotine with the control groups in the worms BZ555 (*p* ≤ 0.005). **(B)** Represents the alleviative effects of nicotine on DA content levels after 6-OHDA treatment. 6-OHDA-intoxicated strains reduced dopamine levels to 63% (*p* ≤ 0.005) compared to untreated control. We observed that nicotine significantly increased dopamine levels at 100 (21%), 150 (45%), and 200 (58%) μM in 6-OHDA intoxicated BZ555 worms dose-dependently. The data represent the mean ± S.D. (*N* = 3). The # hash shows a significant difference between control and 6-OHDA-treated nematodes. While the * asterisk represents a significant difference between 6-OHDA and nicotine-treated groups (*p* ≤ 0.005).

Researchers have demonstrated that animals exposed to 6-OHD exhibit a phenotype of DA neurodegeneration, which subsequently affects dopamine synthesis (Eo et al., 2019). Migration of *C. elegans* is determined by their bodies bending frequently, which determines their speed (Ezcurra et al., 2011; Kress et al., 2014). On NGM media, *C. elegans* could sense food sources, usually bacterial lawns. DA neuronal circuits are responsible for *C. elegans*’ basal slowing rate with food. Consequently, degenerative DA neurons lead to a lower dopamine level, which lowers the ability of nematodes to sense food. Compared with worms treated only with 6-OHDA, the locomotory rate would decrease if nicotine could decrease the 6-OHDA-induced DA neuron degeneration. Nicotine is examined for its ability to save nematodes’ food-sensing behavior under 6-OHDA treatment. As a next step, we examined defects in food-sensing (counted as a “slowing rate”) in 6-OHDA treated worms (BZ555). As compared to control without 6-OHDA treatment, the slowing rate of BZ555 nematodes was decreased 70% (*p* ≤ 0.005), demonstrating DA neuron dysfunction causes a deficit in food-sensing behavior. We observed that nicotine in BZ555 diminished DA neuron degeneration and significantly increased the slowing rate at (100 μM, by 18%), (150 μM, by 49%), and (200 μM, by 86%) (p ≤ 0.005), dose-dependently. These data collectively proved that nicotine could restore 6-OHDA-mediated DA neuron dysfunction Figure 6A.

### 3.6 Nicotine restores dopamine contents in 6-OHDA intoxicated transgenic *Caenorhabditis elegans* strains in a dose-dependent manner

We used an ELISA assay to measure dopamine levels in 6-OHDA and 6-OHDA / nicotine-treated BZ555 strains. We observed that the 6-OHDA-intoxicated strain’s dopamine level was reduced compared to the untreated strain. The dopamine levels in 6-OHDA-intoxicated strains reduced by 63% (*p* ≤ 0.005) compared to untreated control. We observed that nicotine significantly increased dopamine levels by 21, 45, and 58% in 6-OHDA intoxicated BZ555 worms dose-dependently (*p* ≤ 0.005) Figure 6B.

### 3.7 Nicotine reduced *in vivo* ROS generation in a dose-dependent manner

**Figure 7:**
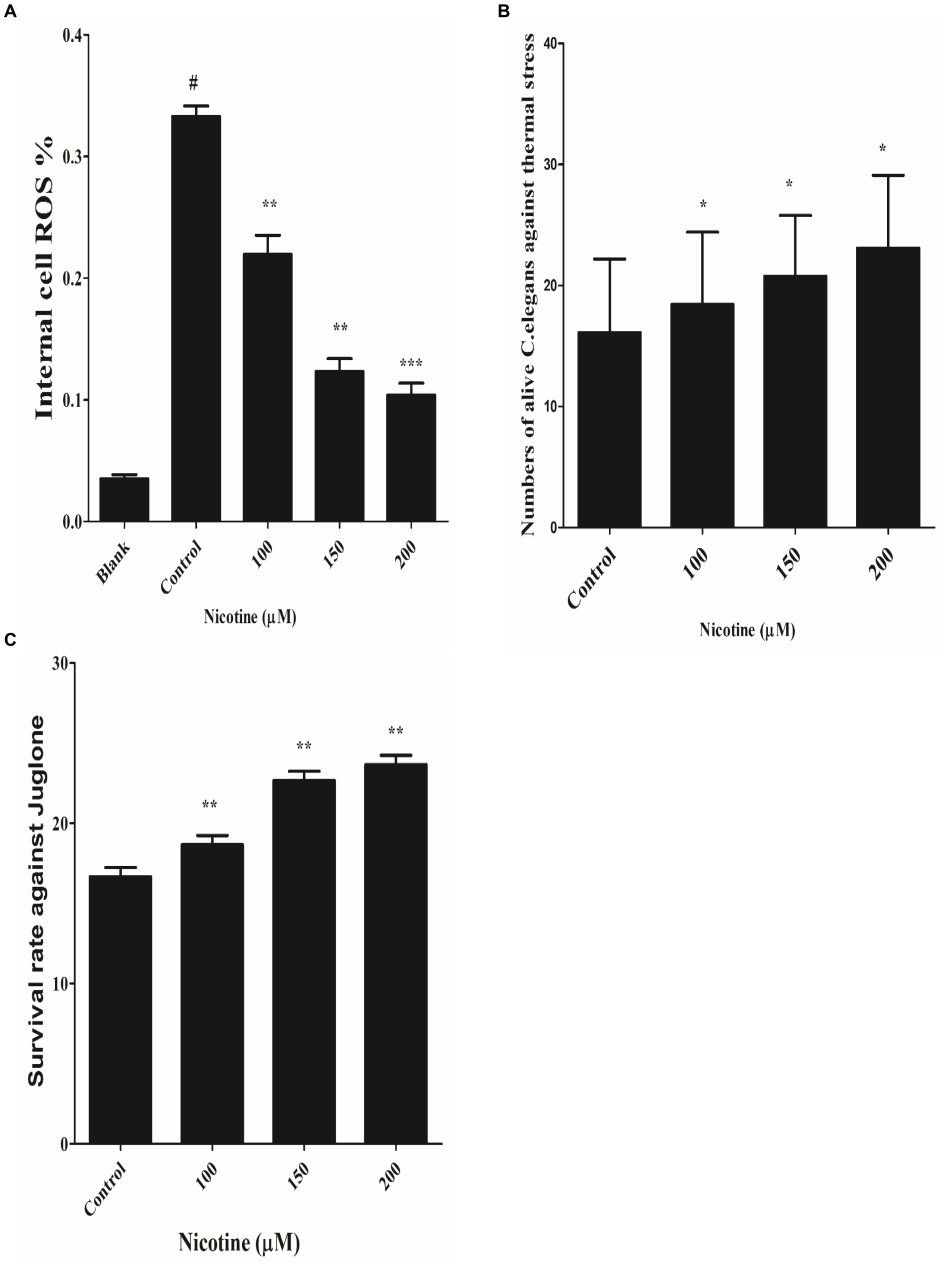
**(A)** Defined the anti-ROS role of nicotine on alive OW13. OW13 was treated with 100, 150, and 200 μM nicotine for 72 h. The samples were then treated with fluorogenic substrate H2DCF-DA in 96-well microtiter plates, and readings were recorded. Results showed that nicotine significantly reduced the ROS production in alive worms up to 100 (34%), 150 (63%), and 200 μM (69%) compared to untreated groups. Data representing the mean ± SD (*n* = 3). (*) depicting the significant difference of (*p* ≤ 0.005) between nicotine and untreated groups. # hash represents significant differences of (*p* ≤ 0.005) between the blank and control group. **(B)** Represents nicotine-significant tolerance against high temperatures of 37°C after 3 h. The result shows that nicotine-treated worms at 100 μM (13%), 150 μM (22%), and 200 μM (30%) survived longer than untreated worms. The data represent the mean ± S.D. (*n* = 3). * An asterisk indicates significant differences (*p* ≤ 0.005) between the nicotine-treated and control samples. **(C)** Represents the nicotine anti-oxidant effect against Juglone in OW13. The result shows that Juglone/nicotine-treated worms at 100 μM (11%), 150 μM (26%), and 200 μM (30%) survived longer than untreated worms (Juglone only). The data represent the mean ± S.D. (*n* = 3).* An asterisk indicates significant differences (*p* ≤ 0.005)between nicotine-treated and control samples.

Per the redox theory of aging, oxidative stress plays a pivotal role in aging (Luceri et al., 2018). A meta-analysis of 47 epidemiologic studies of PD validates the rise of PD incidence with age progression (Collier et al., 2017; Simon et al., 2020). There is also evidence that α-syn aggregation contributes to mitochondrial ROS production (Hsu et al., 2021). An aim of the study was to determine whether nicotine has an antioxidative effect. The ROS labeling dye H2DCF-DA was used to investigate this. Our experimental observation confirmed that nicotine at 100, 150 and 200 μM reduced ROS productions by 34, 63 and 69% dose-dependently as compared to control worms (*p* ≤ 0.005) Figure 7A.

### 3.8 Nicotine-enhanced thermotolerance in transgenic strains of *Caenorhabditis elegans* in a dose-dependent manner

A previous study has shown that nematode thermal stress is relevant to increasing oxidative stress (Koch et al., 2019). In order to investigate the effect of nicotine on thermotolerance, all treated and untreated worms were moved to 37°C for three hours, and the number of live and dead OW13 worms was counted. The nicotine-treated worms survived longer at 100 μM(13%), 150 μM(22%), and 200 μM(30%) than the untreated worms Figure 7B.

### 3.9 Nicotine enhanced the antioxidant capcity in a dose-dependent manner

The effectiveness of a drug under oxidative stress is often related to its ability to extend its life span and to act as an antioxidant (Pandey et al., 2019). It has been shown that worms have an increased antioxidant capacity when their antioxidant system is activated (Shi et al., 2023). We tested the antioxidant capability of nicotine by exposing different treated and untreated BZ555 *C. elegans* to juglone, an oxidative stressor. Juglone/nicotine-treated worms at 100 μM,150 μM, and 200 μM survived longer by 11, 26 and 30% than untreated worms (Juglone only) Figure 7C.

### 3.10 Nicotine-enhanced Daf-16 nuclear translocation in transgenic strains *Caenorhabditis elegans* in a dose-dependent manner

**Figure 8:**
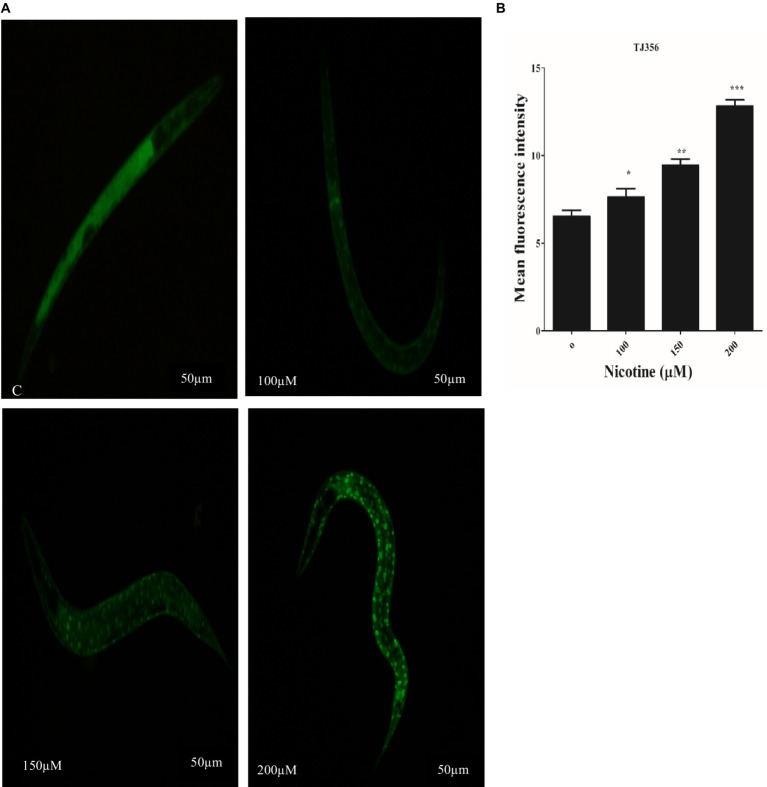
Transgenic strain TJ356 exhibits nicotine’s anti-oxidative capacity through the Daf-16 pathway in a dose-dependent manner. The intensity of GFP in transgenic strain TJ356 is represented by **(A)**. A dose-dependent pattern of Daf-16 nuclear translocation in TJ356 worms treated with nicotine at 100 μM (14%), 150 μM (31%), and 200 μM (49%) is apparent compared to untreated groups. **(B)** Shows a graphical representation of TJ356 fluorescence intensity calculated using image J. The data represent the mean ± S.D. (*n* = 30). Scalebar 50 μm. * An asterisk indicates significant differences (*p* ≤ 0.005) between nicotine-treated and control samples.

Under stress conditions, DAF-16, a *C. elegans* homolog of the forkhead transcription factor (FOXO), is activated and different protective genes are expressed (Link et al., 2016; Zecic and Braeckman, 2020). DAF-16 normally resides in the cytoplasm. In order to activate downstream target genes, activated DAF-16 is transferred from the cytoplasm to the nucleus (Mukhopadhyay et al., 2006). The nuclear localization of DAF-16 can be induced by oxidative stress (Senchuk et al., 2018). TJ356 transgenic worms were used to investigate the effect of nicotine on DAF-16 nuclear translocation. Nicotine-treated worms at 100 μM, 150 μM, and 200 μM enhanced DAF-16 translocation from cytoplasm to nucleus by 14, 31, and 49% in a dose-dependent manner (*p* ≤ 0.005) Figure 8.

### 3.11 Nicotine-enhanced Sod-3 expression in transgenic strains *Caenorhabditis elegans* in a dose-dependent manner

**Figure 9:**
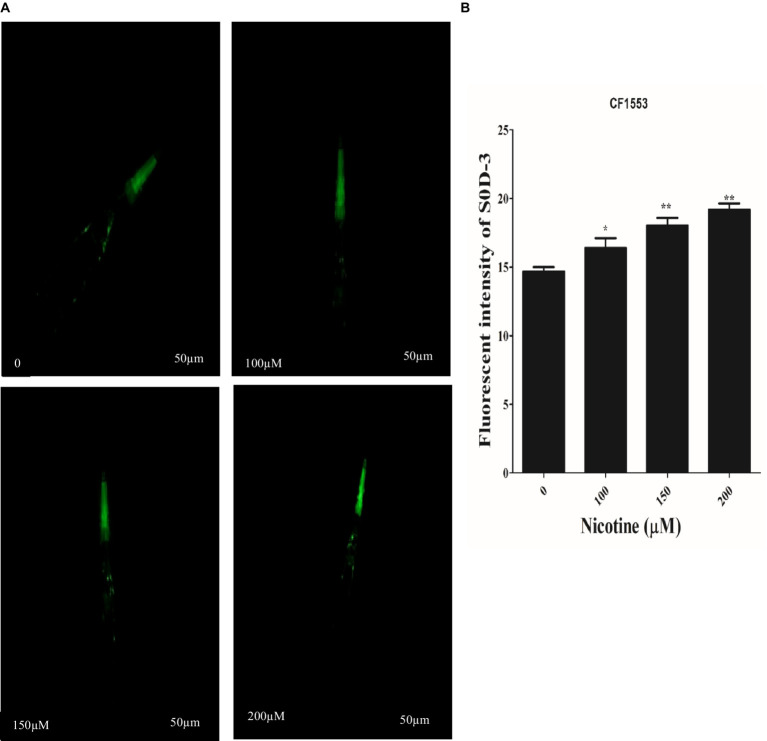
Reporter gene SOD-3::GFP increased anti-oxidant enzymes in transgenic CF1553 worms on nicotine treatment. **(A)** A clear illustration of SOD-3 GFP overexpression in worms treated with nicotine at 100 μM (10%), 150 μM (19%), and 200 μM (23%). **(B)** A graphic representation of SOD-3 reporter gene expression shows that nicotine enhanced antioxidative enzyme actions while reducing oxidative stress, a leading cause of ROS production in Parkinson’s disease. Data were calculated as mean ± SD (*N* = 30). Scale bar 50 μm. * describes a significant difference of *p* ≤ 0.005 between nicotine-treated and untreated groups.

SOD activity plays a key role in scavenging ROS. The high antioxidative properties of a drug contribute to its ROS scavenging capacity (Van Raamsdonk and Hekimi, 2012; Niu et al., 2021). In CF1553 worms, sod-3::gfp encodes superoxide dismutases. The effects of nicotine on the CF1553 worms were determined by treating them with nicotine and determining its antioxidative properties. A dose-dependent increase in SOD-3 expression was found by nicotine in CF1553 worms as compared to control (CF1553 without any drugs) at (100 μM,10%), (150 μM, 19%), and (200 μM, 23%), (*p* ≤ 0.005) Figure 9.

## 4 Discussion

In this study, nicotine was evaluated for its therapeutic efficacy in *C. elegans* models of Parkinson’s. Our research techniques use the benefits of the *C. elegans* model for drug testing, forecasting a drug-specific mode of action comprising easy and exact visualizations of live DA neuron degeneration and α-syn aggregative toxicity. Further, small-scale NGM media culturing of *C. elegans* models significantly reduced the number of drugs needed for examinations. Assays based on *C. elegans* could be influential for rapid assessment, low cost, and screening many novel neuroprotective drugs (Naseer et al., 2021). We assessed nicotine’s optical and toxic concentrations in our *C. elegans* PD models using a food clearance test. Due to the advantages of growing *C. elegans* in *Escherichia coli* liquid cultures and the short life cycle, nicotine was tested at the rate at which the food source OP50 (*E. coli* bacterial strains) was consumed. In an optical observation, worms treated at 250 μM dilution showed toxic behavior, leading to a smaller body size and fewer offspring. This resulted in the death of adults at day intervals and a decrease in food clearance. A concentration of 100, 150, and 200 μM of nicotine added to cultures containing BZ555 and OW13 did not affect food clearance as compared to a concentration of 250 μM (Figure 2).

The 6-OHDA neurotoxin was chosen for this study based on its ability to selectively destroy DA neurons. The transport mechanisms of dopamine allow 6-OHDA to pass in neurons after treatment (Estrada-Valencia et al., 2022). DA neurons degenerate, causing Parkinson’s disease. BZ555 shows PD-like symptoms due to 6-OHDA-induced desensitization of DA neurons (Nass et al., 2002; Kumari et al., 2021). After creating the neurodegenerative model, we treated it with nicotine at 100, 150, and 200 μM concentrations. Compared to control groups, nicotine-recovered degenerated DA neurons dose-dependently (Figure 3). Furthermore, the ELISA assay also revealed that nicotine/6-OHDA treated worms exhibited an increase in DA levels compared to controls (Figure 6B).

The DA neurons control *C. elegans*’ food-sensing behavior (Liao et al., 2020). The uptake of 6-OHDA causes desensitization of DA neurons, which affects functions such as movement and food sensing, which is restored after nicotine treatment (Figure 6A). Nicotine’s antioxidant activity may be responsible for its neuroprotective effects in 6-OHDA neuronal loss, food-sensing behavior defects, and dopamine level declines. The accumulation of protein α-Syn in neuronal cells is another characteristic of Parkinson’s disease (Bérard et al., 2022). Accumulated α-Syn in the muscle walls specifying the *in vivo* toxicity leading to PD-like progressive motility in *C. elegans* (Maulik et al., 2019). In this study, we examined the therapeutic effect of nicotine on α-Synuclein disaggregation in *C. elegans* strain OW13. As a result of the present study, we observed reduced aggregation of α-synuclein and hypothesized that nicotine might promote neuroprotection (Figure 4). There are large quantities of lipid molecules in the CNS, suggesting that their functions are not limited to cell drive or structural component production (Dyall et al., 2022). It is well known that some lipids in the CNS play a vital role in neurotransmission (Xu et al., 2022). Neurodegenerative diseases are caused by defective cell signaling pathways (Hannan et al., 2020). The lipid content of cells modulates all signaling activities (Chen et al., 2020). The altered fatty acid and lipid content levels in Parkinson’s disease concern toxic α-synuclein accumulation through the binding of α-synuclein with lipids (Chalorak et al., 2018; Amri et al., 2022). OW13 worms stained with Nile Red showed significantly higher lipid depositions after nicotine was added (Figure 5).

Our study also found that nicotine reduced α-synuclein accumulation, reduced lipid peroxidation, and restored lipid levels. Thus, such protective effects could maintain efficient cellular signaling by regulating lipid arrangement disturbances. Both sporadic and familial forms of Parkinson’s disease are thought to be caused by oxidative stress (Chang and Chen, 2020). A moderate level of oxidative stress can therefore lead to cellular reactions and neuronal cell death (Tchekalarova and Tzoneva, 2023). In epidemiologic studies of PD, age progression and oxidative stress are associated with higher PD incidence (Collier et al., 2017). Nicotine reduced *in vivo* ROS levels dose-dependently and protected neurons against oxidative stress (Figure 7A). *C*. elegans’ oxidative stress increases with thermal stress (Koch et al., 2019). The survival rate of nicotine-exposed worms was higher than untreated worms in a dose-dependent manner (Figure 7B). The naphthoquinone juglone is one of the most abundant naphthoquinones, belongs to the quinone class, and produces hydrogen peroxide and intracellular superoxide. As a result, biological macromolecules are damaged, and the aging process and diseases are accelerated (Benites et al., 2018; Ramos Boldori et al., 2023). Our worms were treated with Juglone, an intracellular ROS generator, to validate nicotine’s effect against oxidative stress. Nicotine treated worms have a higher survival rate under stressed conditions than untreated worms (Figure 7C).

Stress tolerance and lifespan extension are well-known functions of DAF-16, a downstream transcription factor of the IIS pathway (Zhu et al., 2023). Inhibition of insulin/IGF receptor DAF-2 reduces the activity of phosphoinositide 3-kinase (PI3K)/Akt kinase cascade, which, in turn, dephosphorylates and activates DAF-16/FOXO transcription factor through enhanced translocation from the cytoplasm to the nucleus, regulating genes involved in stress resistance and longevity (Sun et al., 2021). Nicotine promoted the translocation of DAF-16 to the nucleus, activating detoxification and defense proteins in the nematode. A higher fluorescence was observed in nicotine-treated TJ356 than in untreated cells, suggesting that this factor is translocated from the cytoplasm to the nucleus (Figure 8). Superoxide dismutase (SOD-3) is an antioxidant enzyme that reduces ROS to prolong life (Balestrino and Schapira, 2019). PD is a neurodegenerative disorder associated with aging. An important risk factor for PD is mitochondrial ROS-induced oxidative stress (Kung et al., 2021). The *C. elegans* genome contains five superoxide dismutase (SOD) genes. The antioxidant enzyme SOD-3 reduced oxidative stress by reducing ROS and contributed to a longer lifespan (Li et al., 2021). Therefore, we assessed the active role of nicotine in promoting SOD-3 activity in CF1553 *C. elegans*. Nicotine treatment at 100, 150, and 200 μM concentrations exhibited a significant increase in SOD-3 expression compared to untreated groups (Figure 9). In our study, nicotine demonstrated antioxidative properties and reduced ROS levels in worms. Based on the tests above, nicotine is probably associated with neuroprotective effects due to its anti-radical properties. Future research will be needed to determine the precise mechanism behind these results.

## 5 Conclusion

Nicotine’s neuroprotective effects were assessed in pharmacological transgenic PD models. According to our assessment, nicotine augmented neuroprotection in 6-OHDA-treated nematodes and reduced α-synuclein accumulation—furthermore, our drug increased lipid deposition, food sensing behavior, SOD-3, and Daf-16 fluorescence. Based on various assays, nicotine exhibited excellent antiparkinsonian properties. Compounds like this are readily available, inexpensive, and highly effective ways to regulate DA neurons. It is the first report examining nicotine’s anti-parkinsonism properties in *C. elegans* PD models. Further research will be required to determine the precise mechanism behind these results.

## Data availability statement

The original contributions presented in the study are included in the article/Supplementary material, further inquiries can be directed to the corresponding author.

## Ethics statement

The manuscript presents research on animals that do not require ethical approval for their study.

## Author contributions

IU: Conceptualization, Formal analysis, Investigation, Methodology, Software, Writing – original draft, Writing – review & editing. LZ: Formal analysis, Methodology, Software, Writing – review & editing. SU: Data curation, Formal analysis, Software, Writing – review & editing. YZ: Writing – review & editing, Conceptualization, Validation, Resources, Data curation. XW: Conceptualization, Data curation, Investigation, Project administration, Resources, Supervision, Writing – review & editing. HL: Conceptualization, Funding acquisition, Investigation, Project administration, Resources, Supervision, Validation, Visualization, Writing – review & editing.
